# The Dorsal Visual System Predicts Future and Remembers Past Eye Position

**DOI:** 10.3389/fnsys.2016.00009

**Published:** 2016-02-24

**Authors:** Adam P. Morris, Frank Bremmer, Bart Krekelberg

**Affiliations:** ^1^Neuroscience Program, Department of Physiology, Biomedicine Discovery Institute, Monash UniversityClayton, VIC, Australia; ^2^Department of Neurophysics, Philipps-Universität MarburgMarburg, Germany; ^3^Center for Molecular and Behavioral Neuroscience, Rutgers UniversityNewark, NJ, USA

**Keywords:** eye movements, posterior parietal cortex, vision, electrophyisology, population codes, decoding

## Abstract

Eye movements are essential to primate vision but introduce potentially disruptive displacements of the retinal image. To maintain stable vision, the brain is thought to rely on neurons that carry both visual signals and information about the current direction of gaze in their firing rates. We have shown previously that these neurons provide an accurate representation of eye position during fixation, but whether they are updated fast enough during saccadic eye movements to support real-time vision remains controversial. Here we show that not only do these neurons carry a fast and accurate eye-position signal, but also that they support in parallel a range of time-lagged variants, including predictive and post dictive signals. We recorded extracellular activity in four areas of the macaque dorsal visual cortex during a saccade task, including the lateral and ventral intraparietal areas (LIP, VIP), and the middle temporal (MT) and medial superior temporal (MST) areas. As reported previously, neurons showed tonic eye-position-related activity during fixation. In addition, they showed a variety of transient changes in activity around the time of saccades, including relative suppression, enhancement, and pre-saccadic bursts for one saccade direction over another. We show that a hypothetical neuron that pools this rich population activity through a weighted sum can produce an output that mimics the true spatiotemporal dynamics of the eye. Further, with different pooling weights, this downstream eye position signal (EPS) could be updated long before (<100 ms) or after (<200 ms) an eye movement. The results suggest a flexible coding scheme in which downstream computations have access to past, current, and future eye positions simultaneously, providing a basis for visual stability and delay-free visually-guided behavior.

## Introduction

The primate visual system makes use of the exquisite sensitivity of the fovea by continually directing the eye toward new areas of interest. As a consequence of this active strategy, visual information must be combined with up-to-the-moment information about eye (and head) position to make sense of the environment (Soechting and Flanders, 1992). This additional information allows the brain to take into account self-induced changes in the retinal image and to construct stable representations of visual space—a prerequisite for goal-directed behavior (e.g., reaching, or avoiding collision during self-motion). In previous work, we have argued that suitable eye position signals (EPS) are available in the middle temporal (MT), medial superior temporal (MST), ventral intraparietal (VIP), and lateral intraparietal (LIP) areas of the posterior parietal cortex (PPC) (Bremmer et al., 1997a,b, 1999; Duhamel et al., 1997; Boussaoud and Bremmer, 1999; Schlack et al., 2005; Morris et al., 2012, 2013).

The instantaneous eye position, however, is only one aspect of eye position that is of interest to active vision. Several well-known peri-saccadic phenomena would benefit from easy access to past, current, and future eye positions. For instance, before executing a saccade, the visual system could use information on the future eye position to remap visual information from neurons currently receiving input from a specific spatial location to those receiving input from that location after the saccade (Duhamel et al., 1992; Morris et al., 2007; Schneegans and Schöner, 2012; Ziesche and Hamker, 2014). Similarly, the comparison of visual input before and after a saccade that may contribute to perceptual stability across saccades could benefit from knowledge of the eye position before and after that saccade (Prime et al., 2011; Crapse and Sommer, 2012). In addition, future eye-position signals could be used to suppress the activity of (subsets of) visual neurons during the saccade (a potential neural correlate of saccadic suppression (Ibbotson and Krekelberg, 2011), while boosting the activity of neurons that will process visual information near the saccade target (a potential neural correlate of shifts in attention (Ziesche and Hamker, 2011; Zirnsak et al., 2011, 2014). In general, any form of transsaccadic integration of information would seem to require access to a combination of current, past, and future eye positions. Here we asked whether such flexible eye-position signals are available in LIP, VIP, and MT/MST.

In the one area where multiple groups have studied the peri-saccadic dynamics of eye position signals (LIP), the results have been quite contradictory. We showed that the EPS in LIP is accurate and precise during fixation (Morris et al., 2013), but for brief periods around saccades, the signal first leads, and then lags the true eye position (Morris et al., 2012). This mismatch is consistent with errors in localization that occur around the time of saccades (Honda, 1991; Dassonville et al., 1992; Cai et al., 1997; Lappe et al., 2000). Xu et al. (2012), however, reported that the EPS in LIP lagged behind the eye at the time of saccades by around 150 ms. Graf and Andersen (2014), finally, showed that the population activity in LIP contained information to accurately classify the past, current, and future eye position (contradicting the Xu et al. result). Moreover, this accuracy was not reduced at the time of saccades as one might expect if eye position inaccuracy were related to peri-saccadic mislocalization as we claimed (Morris et al., 2012). We will return to these issues in the discussion, but in our view these discrepancies among studies arise primarily due to limited sampling of neuronal populations, and a focus on the limited information carried explicitly by single neurons (Xu et al., 2012) vs. the rich information that can be extracted from populations of neurons (Morris et al., 2013; Graf and Andersen, 2014).

In this contribution we test the hypothesis that the dorsal visual system carries in parallel a continuum of time-lagged, continuous eye-position signals, including anticipatory, zero-lag, and delayed signals. We predicted that these EPS are represented in a distributed fashion across the neurons in areas LIP and VIP in the intraparietal sulcus, and areas MT and MST in the superior temporal sulcus. Our hypothesis is built on the idea that even if inputs that explicitly encode eye position are slow to update (Xu et al., 2012), the influences of corollary discharge (e.g., suppression, enhancement, etc. Duhamel et al., 1992; Bremmer et al., 2009; Ibbotson and Krekelberg, 2011) may provide sufficient information about impending eye movements to predict future/past eye positions. Similar ideas have been proposed in modeling studies to account for predictive remapping of visual activity during saccades in area LIP (Schneegans and Schöner, 2012; Ziesche and Hamker, 2014).

To test this hypothesis, we developed a novel approach in which we construct a linear decoder whose output provides a metric representation of eye position, and is computed as a weighted sum of instantaneous firing rates in a recorded sample of neurons. The pooling weights are chosen to approximate a specific desired output (e.g., a synthetic EPS that leads the actual eye) and the performance of the decoder is quantified using an independent set of experimental trials (i.e., in cross-validation).

The main difference with the decoding approach used by Graf and Andersen (2014) is that our linear decoder generates a continuous, metric representation of eye position, not just a categorical estimate of the most likely eye position. One can think of this linear decoder as a convenient theoretical construct that quantifies whether certain information (e.g., the eye position some 200 ms previously) is reliably present in the recorded cells. Alternatively, one can view this construct as an über-neuron that could reasonably exist in the brain, but the experimenter's electrode happened not to get near it in this particular study. We return to this in the Discussion.

We first analyzed how firing rate changes over time at the time of saccades in darkness. Consistent with the results of Xu et al. (2012), the neural dynamics of individual neurons matched neither future, nor true, nor past eye position reliably. Using our über-neuron analysis, however, revealed that the distributed patterns of activity across many neurons provided a highly flexible source of information about eye position. Specifically, we found reliable estimates of future eye position starting ~100 ms before saccade onset and reliable memories of past eye position up to ~200 ms after a saccade.

## Materials and methods

The current study consists of a re-analysis of electrophysiological data reported previously (Morris et al., 2012, 2013). Experimental and surgical procedures are described in full in Morris et al. (2012) and Bremmer et al. (2009), were performed in accordance with published guidelines on the use of animals in research (European Council Directive 86/609/EEC and the National Institutes of Health Guide for the Care and use of Laboratory Animals), and approved by local ethics committees (Regierungspräsidium Arnsberg, Ruhr-Universität Bochum).

### Electrophysiology

We recorded single unit action potentials extracellularly using single tungsten-in-glass microelectrode penetrations through the intact dura. Recordings were made in four regions across a total of four hemispheres in two macaque monkeys, including the LIP and VIP areas of the PPC, and the MT and medial temporal (MST) areas. LIP and VIP were recorded within the same hemisphere and MT and MST in the other, in opposite left-right configuration across the two animals. We report the results from all neurons for which we recorded at least 6 trials per experimental condition. A total of 276 neurons were analyzed, including 74 from area LIP, 107 from VIP, and 95 from areas MT and MST combined.

### Behavior

The animal was seated in a primate chair facing a translucent screen (60° × 60° of visual angle) in near darkness and performed an oculomotor task for liquid reward. The animal's head was stabilized using a head-post and eye position was monitored using scleral search coils. The fixation dots were small, faint light-emitting diodes back-projected onto the screen (0.5° diameter, 0.4 cd/cm2). Each trial of the animal's task began with fixation on a dot for 1000 ms. The dot then stepped either rightward or downward by 10° (with equal probability), cueing the animal to perform a saccade to the new position and hold fixation for a further 1000 ms. The initial position of the fixation dot was selected pseudorandomly across trials from five possible locations arranged like the value 5 on a standard six-sided die ([*x*,*y*] = [0,0],[−10,10],[10,10],[10,−10],[−10,−10]). Trials in which the animal failed to maintain gaze within 1° of the dot position during fixation intervals or to perform the saccade within 500 ms of the cue to move were terminated without reward (and not analyzed).

### Data analysis

All analyses were performed in MATLAB R2014b (The MathWorks, Inc.). The raw data included timestamps for the recorded spikes for each neuron and eye position data. Spike-times within each trial were expressed relative to the onset of the ~10° amplitude primary saccade (detected offline using eye velocity criteria), and converted to instantaneous firing rates using a 50 ms wide counting window stepped in 25 ms increments from −800 ms to +800 ms. These firing rates were then averaged over trials separately for each of the 10 task conditions (five initial fixation positions and two saccade directions). The data for the five initial positions were then averaged to yield a single firing rate time course for each of the two saccade directions (rightward and downward) for each neuron. For each cortical area, the time courses for the two saccade directions were compiled into matrices, *R*_*rwd*_ and *R*_*dwd*_, in which the number of rows was equal to the number of time points and the number of columns was equal to the number of neurons.

#### Principal component analysis

We used principal component analysis (PCA) to investigate whether a small number of typical firing rate modulation patterns could capture the observed neural dynamics at the time of saccades. As we were particularly interested in saccade-direction specific dynamics, we first subtracted the response time course for rightward saccades (*R*_*rwd*_) from the time course for downward saccades (*R*_*dwd*_). The resulting differential time courses (a matrix in which each row represents a time point, and each column a neuron) were subjected to PCA. This analysis extracts an ordered set of time courses; the first few of these time courses serve as a reduced basis to describe a large fraction of the variance in the full complexity of the saccade direction specific changes in firing rate in the population.

#### Linear decoding

**Figure 1:**
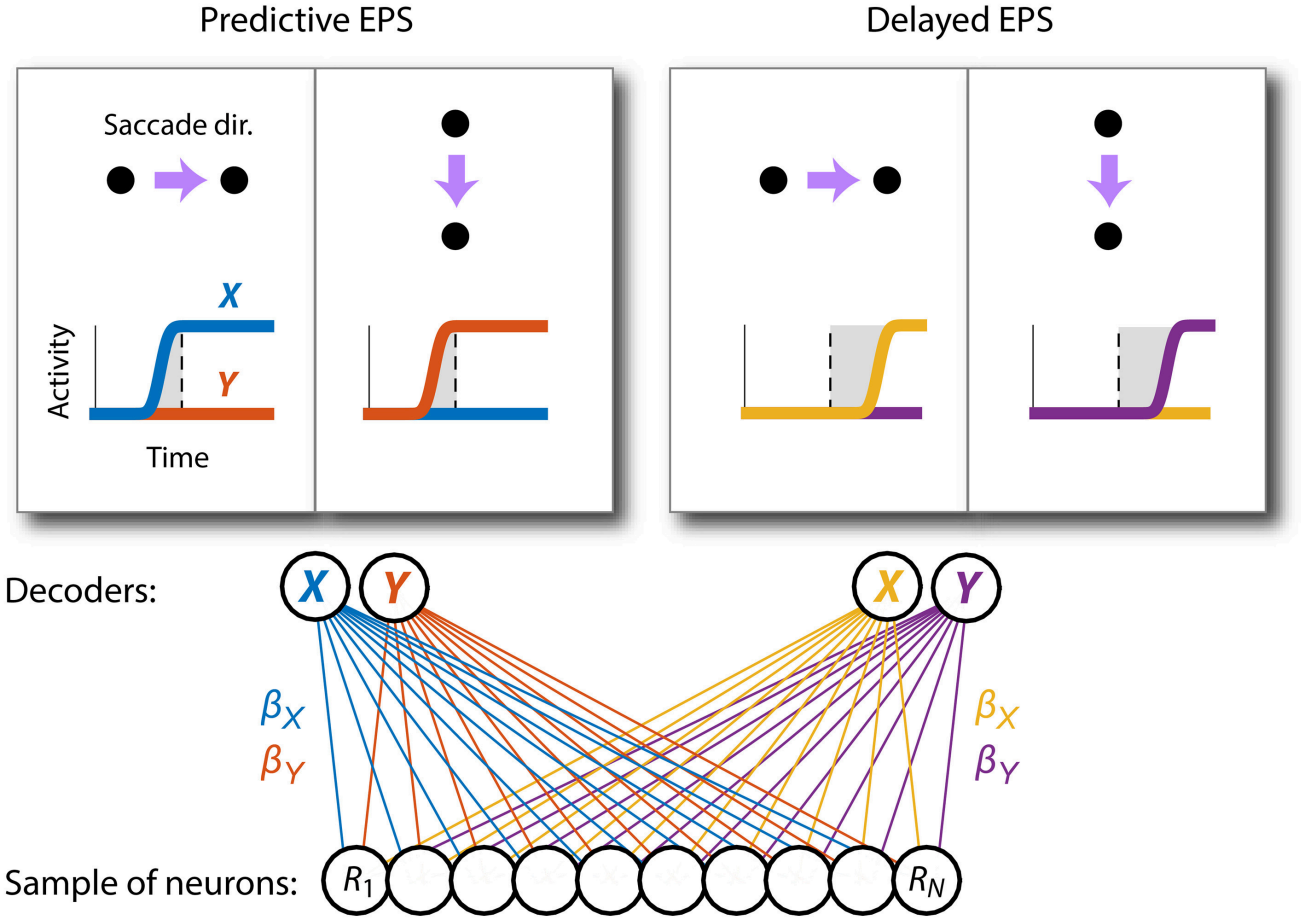
**Selective pooling of neural activity generates time-lagged eye-position signals**. Two über-neurons ($\hat{X}$ and $\hat{Y}$) were constructed to represent the horizontal and vertical components of eye position through their firing rates. Each took a weighted sum (with weights, β_*X*_ and β_*Y*_, respectively) of activity (R) across a sample of experimentally observed cortical neurons (circles). Idealized time courses for these decoder units are shown for two orthogonal saccade directions (rightward and downward). Using one set of weights (blue and red), $\hat{X}$ and $\hat{Y}$ carry a predictive representation of eye movements (the vertical dashed line indicates the actual onset of eye movement). Using a different set of weights (yellow and purple), their representation lags behinds the actual eye. Both time courses were decoded from the same spatiotemporal patterns of neural activity in the population. (Note that for the purpose of visualization, the downward saccade in this and all other figures is plotted as if it was upward).

The aim of our main analysis was to determine whether the activity of the recorded neurons could be combined into a pair of output variables, $\hat{X}\left(t\right)$ and $\hat{Y}\left(t\right)$, that would mimic the animal's true horizontal and vertical eye position over time (Figure 1). These estimated eye positions were computed by taking weighted sums of activity across the population of neurons at each point in time:

(1) $$\begin{array}{l} \hat{X}\left(t\right)=\;R\left(t\right)\beta_{X}+c_{X}\\ \hat{Y}\left(t\right)=\;R\left(t\right)\beta_{Y}+c_{Y} \end{array}$$

where β is a column vector of weights (one number per neuron) and *c* is constant. One can interpret $\hat{X}$ and $\hat{Y}$ as firing rates of two downstream neurons that represent the eye position explicitly in their firing rate. We call these neurons “über-neurons.” In this case β represents the (unitless) strength of the synapse connecting each recorded neuron to the über-neuron and *c* its spontaneous firing rate. Alternatively, one can view $\hat{X}$ and $\hat{Y}$ as abstract representations of linearly decoded eye position information (in degrees of visual angle [°]) present in the recorded population. In this interpretation β has units of °/spike and *c* has units of degrees.

We use matrix notation to link these equations to the data. Using the horizontal channel as an example, we modeled the relationship between the eye's true position and firing rates as:

(2) $$\begin{array}{l} X=R\beta_{X}+\varepsilon \end{array}$$

where ε represents additive noise. For rightward saccades, the target of the regression (*X*_*rwd*_) was approximated by a scaled cumulative Gaussian that closely matched the spatiotemporal profile of the average saccade (mean; μ = 25 ms, standard deviation σ = 10). For downward saccades, which contained negligible horizontal displacement, the target eye position (*X*_*dwd*_) was set to 0 for all time points. Analogous target representations were used for the vertical coordinate of the eye (Y).

The design matrix, *R*, is a two dimensional matrix representing the firing rate of each neuron (columns) recorded at a specific time relative to the saccade (rows). An additional column of ones was included to represent the constant offset in the linear model (*c* in Equation 1).

Equation 2 is therefore a set of linear equations with unknown parameters β_*X*_. This column vector represents the contribution of each neuron to the horizontal eye position. The last entry in this vector represents the constant offset (*c*_*X*_). Importantly, although the weights were different for $\hat{X}$ and $\hat{Y}$ (Equation 1), *they were fixed across the two saccade directions*; that is, the decoder had to use the same read-out for eye position irrespective of saccade direction. This is an important criterion for a useful eye-position signal. β_*X*_ was therefore estimated simultaneously across both saccade directions by concatenating the regression targets (*X*_*rwd*_ and *X*_*dwd*_) in time and treating them as a single time course. The design matrices (*R*_*rwd*_ and *R*_*dwd*_) were concatenated in the same way.

To estimate β_*X*_, we selected half of the experimental trials randomly (train set) from each of the 10 task conditions for each neuron to construct *R* and then solved the linear system using a regularized form of regression (“ridge regression”; using the “ridge” function in MATLAB). Ridge regression encourages sparseness in the read-out by penalizing the decoder for large regression coefficients (Martinez and Martinez, 2007). Specifically, the objective function that was minimized to estimate β_*X*_ included not only the standard cost term (i.e., sum of squared errors), but also an additive penalty term, $\gamma \overset{N}{\underset{i=1}{\sum }}\beta_{i}^{2}$; that is, the sum of the squared pooling weights across all neurons, scaled by a shrinkage parameter (set to 30 for all results reported here, chosen empirically by testing a wide range of values [0–100] and noting the smallest value at which cross-validation performance stabilized). This regularization approach reduced variance in decoder estimates by preventing over-fitting arising from colinearity among predictor variables (in this case, among responses across neurons).

After estimating β_*X*_, we used the remaining trials (test set) to construct a new *R* matrix, and generated predicted eye positions ($\hat{X}$) by evaluating Equation 1. This use of independent data for β_*X*_ estimation and $\hat{X}$ prediction ensures that the results reflect reliable aspects of neural representation in cortex and not the exploitation of noise to fit the target signal. To obtain estimates of reliability, we repeated this cross-validation process 1000 times by designating new random subsets of trials as train and test sets. Data figures throughout this paper show the mean and standard deviation of these estimated eye positions across cross-validation sets.

#### Time-lagged eye position signals

To determine whether a neural population could support predictive or delayed representations of the actual eye movement, the general linear model analysis was repeated using a range of time-lagged eye signals as the target variable for the regression. Specifically, the mean (μ) of the cumulative Gaussian used to model eye position (*X* and the analogous *Y* in the estimation step of Equation 2) was varied to generate lags from −400 ms to +400 ms in 100 ms steps. Negative and positive lag values correspond to predictive and delayed signals respectively. Herein, we refer to the temporal offset between the target signal and the actual eye as the “target lag.” Note that these time-shifting operations were applied only to the target signal for the regression, not the neural data.

#### Quantification of achieved signal lags

The linear readout typically generated sigmoid-like representations of eye position over time. If the output were perfect, the times of these sigmoidal transitions would have matched those of the target signals—that is, the “achieved lag” would match the target lag. To quantify the achieved lag for each target lag condition, the mean outputs of the $\hat{X}$ and $\hat{Y}$ units over all cross-validation sets were fit with a cumulative Gaussian function (using “lsqcurvefit” in MATLAB) that had four free parameters (mean, standard deviation, amplitude, and vertical offset). These parameters were estimated simultaneously across both saccade directions by accumulating fit error (sum of squared residuals) across the two saccade channels (i.e., the $\hat{X}$-unit for rightward saccades, and the $\hat{Y}$-unit for downward saccades). The difference between the fitted μ parameter and that of the zero-lag condition [i.e., 25 ms (because data were aligned to saccade onset)] represented the achieved lag. The slope (σ) provided a measure of signal velocity, which we converted to a measure of saccade duration based on the interval between the 1st and 99th percentile of the Gaussian. The variance of these parameters was estimated by repeating the sigmoid fit to each of the 1000 cross-validation sets. In those cases, we constrained the optimization by fixing the amplitude and offset parameters to their values from the fit to the mean across cross-validation sets and estimated only the mean and slope.

#### Sparseness and weight analysis

To analyze how much each recorded neuron contributed to the decoding performance, we first determined, for each target lag, and separately for $\hat{X}$ and $\hat{Y}$, the mean weights across cross-validation sets. We restricted our analysis to target lags that were well captured within each cortical area, defined as a total *R*^2^ of greater than 0.75 for the peri-saccadic epoch (i.e., −100 ms through to +200 ms for LIP and VIP; −100 ms through to +100 ms for MT/MST; see **Figure 7**). [The validity of examining *mean* weights rests on an assumption that the decoder from those weights provides a good fit to the data. We confirmed that this was the case for all cortical areas and across these target lags (all *R*^2^ > = 0.87)].

We defined a neuron's “pooling weight” as the average of its absolute weight values to $\hat{X}$ and $\hat{Y}$. These aggregate pooling weights were normalized such that their sum was equal to one across the population. A pooling weight of zero indicates a neuron that is not used by any of the über-neurons, whereas a weight of 0.05 indicates a neuron that contributes 5% of the total input to the über-neurons. For **Figure 9** we constructed a histogram across the sample of recorded neurons for each cross-validation set and then averaged the bin values across sets and target lags. These mean histograms provide insight into the sparsity of the code.

## Results

We recorded the spiking activity of neurons in macaque areas LIP, VIP, MT, and MST during an oculomotor task consisting of rightward and downward saccades. We have shown previously for this data-set that many neurons in all four areas show tonic changes in firing rate across changes in eye position (Morris et al., 2012, 2013). In addition, however, almost all neurons, including those without significant eye position effects, showed modulations of neural activity around the time of the saccades. The dynamics of these changes varied greatly across neurons and in many cases depended on the direction of the saccadic eye movement, as we show next.

### Population dynamics

**Figure 2:**
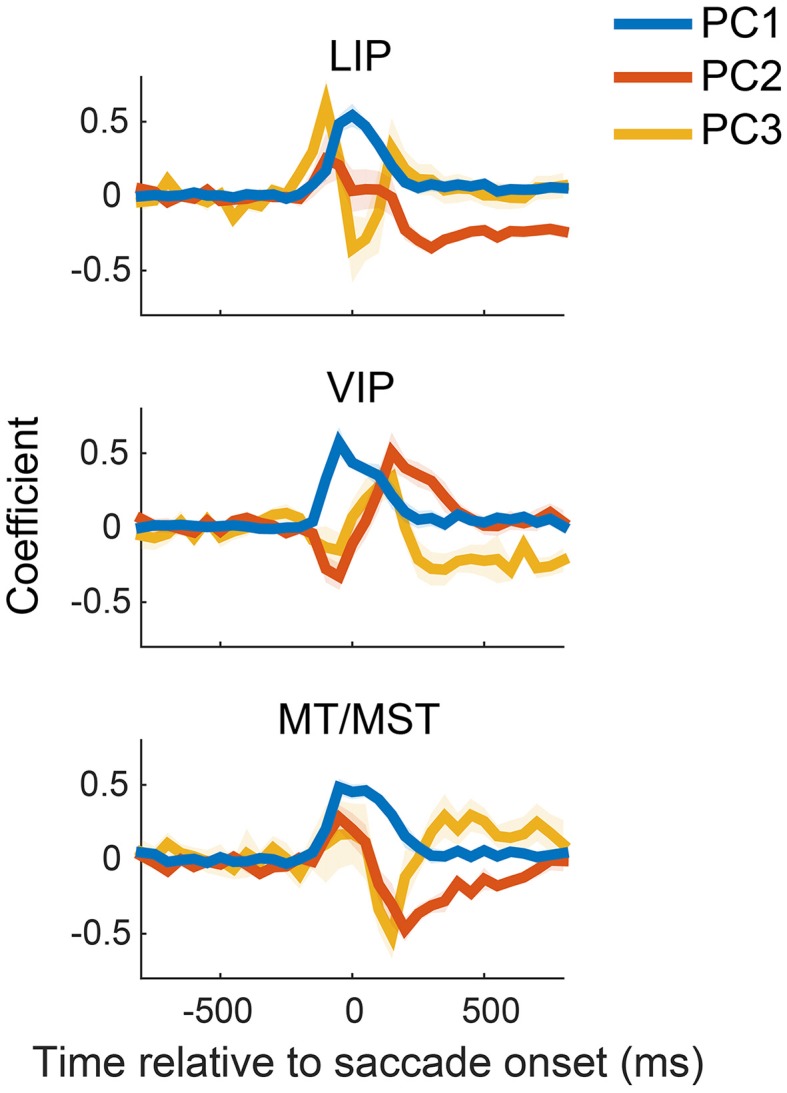
**Principal component analysis of neural activity related to saccade direction**. The first three components (PC 1-3) for each of the cortical areas are plotted relative to saccade onset. Components are ordered by decreasing contribution to the total variance (i.e., PC1 explained the most variance). These components reveal a variety of peri-saccadic effects of neural activity, including broad enhancement and suppression and biphasic modulations. The shaded regions represent standard errors, and were obtained by repeating the PCA on 1000 bootstrapped samples of neurons (i.e., resampling neurons). LIP, lateral intraparietal area; VIP, ventral intraparietal area; MT/MST, middle temporal and medial superior temporal areas.

To illustrate the diversity of peri-saccadic dynamics across neurons, we performed a PCA on the saccade direction specific components of the firing rate (see Materials and Methods), separately for each cortical region (MT and MST neurons were pooled). The first three principal components for each cortical area are shown in Figure 2 and together accounted for 75, 66, and 70% of the variance across neurons for areas LIP, VIP, and MT/MST, respectively. This implies that the typical time courses of these neurons can be described as a linearly weighted combination of the curves shown in Figure 2.

The components revealed complex dynamics underlying the firing rates of these neurons. The first component, for example, consisted of a broad enhancement or reduction of activity for all three cortical regions. Because we analyzed the saccade direction-specific time courses, this corresponds to an enhancement/reduction for one saccade direction relative to the other (and is therefore complementary to the general changes in peri-saccadic firing rate discussed in Bremmer et al., 2009). The deflections began shortly before (≈−100 ms) the onset of the saccade and did not stabilize until roughly 150 ms after the eye landed at the new fixation position. The scores for this component (i.e., the weight of a component for a neuron, or, equivalently, the projection of a neuron's time course on the relevant component) were distributed across positive and negative values (data not shown), suggesting an approximate balance between enhancement and reduction for rightward and downward saccades in our sample. A second prominent component showed a sustained difference before and after the saccade (e.g., PC2 in LIP, PC3 in VIP and MT/MST); intuitively such a component is necessary to carry sustained eye position information during fixation.

The PCA analysis serves mainly to illustrate the richness of the response time courses in the population. Next we turn to our population decoding approach, in which such richness is not a sign of “inconsistent behavior” (cf. Xu et al., 2012) but the basis for a flexible neural representation.

### A flexible representation of eye position

We used a linear decoding approach to reveal information about eye position and eye movements in the recorded neural data (Figure 1). Two artificial downstream units were constructed: one to represent the horizontal coordinate of the eye (X), and another to represent the vertical component (Y). The firing rates of these units were assumed to be isomorphic with eye position, where 1 spike/s was equivalent to 1° of eye rotation. We refer to these units as über-neurons.

Each über-neuron took a weighted sum of the recorded neural activity from a given cortical area. In a first analysis, the weights were optimized such that the predicted eye positions represented by the output—$\hat{X}$ and $\hat{Y}$—approximated the true spatiotemporal dynamics of the eye (i.e., the fixation-saccade-fixation sequence of the behavioral task). The optimal weights were estimated from 50% of the trials and tested in cross-validation with the remaining 50% of trials (see Materials and Methods). Importantly, *weights were fixed over time and across both saccade directions*. Accordingly, the only factor that could lead to changes in the outputs of $\hat{X}$ and $\hat{Y}$ over time and across conditions was a change in the activity of the recorded neurons.

**Figure 3:**
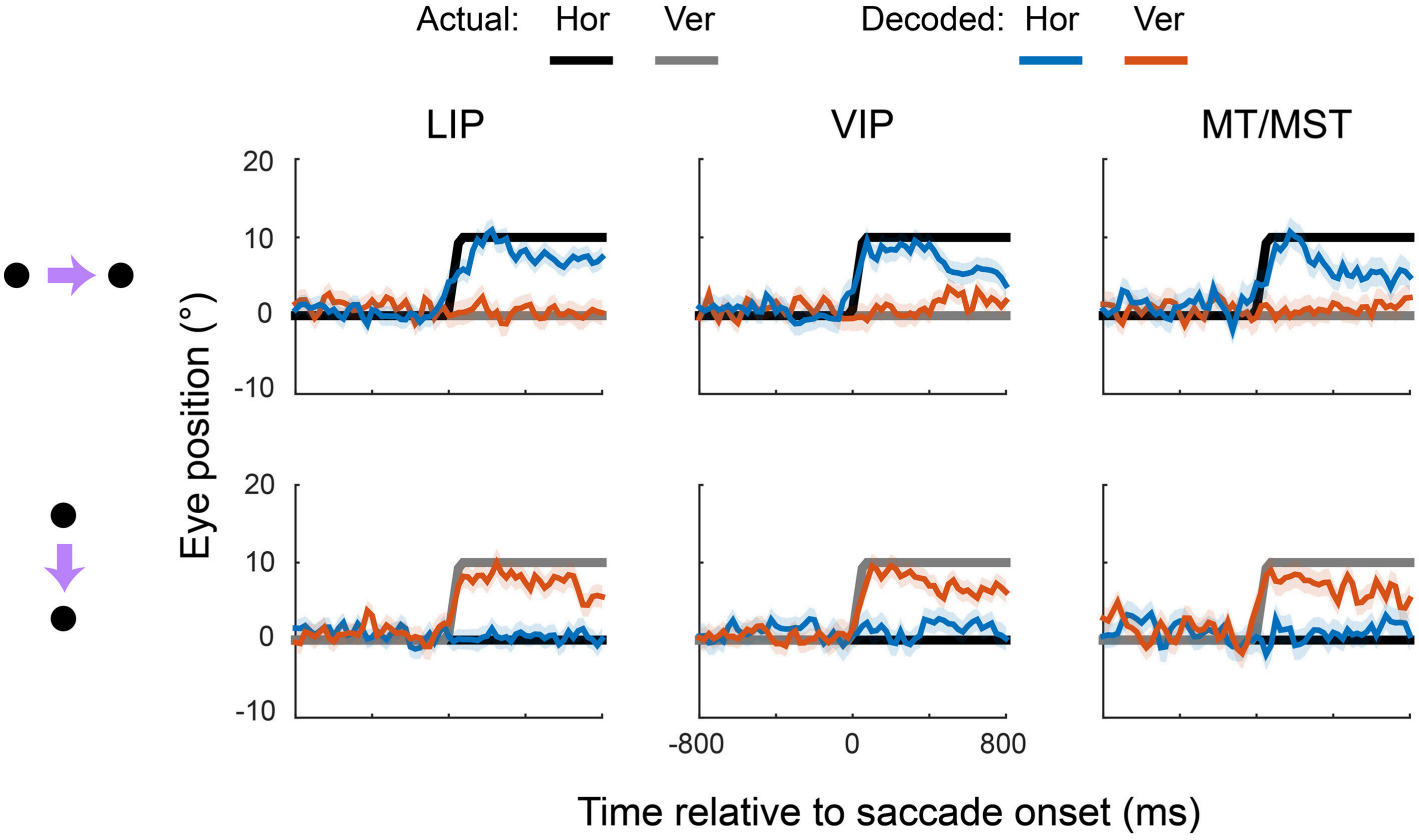
**The dorsal visual system supports accurate, zero-lag representations of eye position**. Each plot shows the output of the X (blue) and Y (red) decoders over time, plotted relative to saccade onset. Data are plotted as eye position in degrees of visual angle for direct comparison with the true eye (black and gray), but can equally be considered to be firing rates of the über-neurons. The top and bottom rows correspond to rightward and downward saccades, respectively. The same pooling weights were used for the two saccade directions, so the different observed time courses reflect only changes in the underlying activity of the recorded neurons. The plotted values and shading are the means and standard deviations, respectively, of the decoder output across all cross-validation test sets.

Figure 3 shows the output of the $\hat{X}$ (blue) and $\hat{Y}$ (red) units for rightward and downward saccades, plotted separately for each cortical area. For comparison, the true eye position is shown (black and gray). The linear read-out provided a good match to the spatiotemporal profile of the eye for all three cortical regions. The $\hat{X}$ unit, for example, showed a sigmoid-like time course during rightward saccades but remained relatively constant during downward saccades. The $\hat{Y}$ unit showed the opposite pattern.

Coefficients of determination (*R*^2^) indicated that the linear predictions ($\hat{X}$ and $\hat{Y}$) accounted for 85, 80, and 71% of the variance in true eye position (over time, and across saccade directions) for areas LIP, VIP, and MT/MST, respectively. Moreover, the timing and velocity of the transitions from the pre-saccadic to post-saccadic positions were close to those of the actual eye movement. Zooming in on the peri-saccadic interval (i.e., by calculating fit error only within 100 ms of saccade onset and offset), *R*^2^ values were 84, 89, and 83% for areas LIP, VIP, and MT/MST, respectively. There was, however, some degradation of the representation late into the second fixation interval, starting ~400 ms after the saccade, more so in areas VIP and MT/MST than in LIP. This is in contrast to our previous finding, where we showed that an accurate representation of the eye is available at this time (using a non-linear decoding approach, optimized for the fixation interval).

### Time-lagged representations of eye movements

The results presented in Figure 3 show that an accurate representation of the eye during fixation and across saccades is available in the population activity of neurons in areas LIP, VIP, and MT/MST. This information can be read out by downstream units within a single (i.e., monosynaptic) computational step. Next we examined whether the same neural populations could in parallel support representations of the eye that were updated in advance of the actual eye—that is, predictive eye-position signals like that shown in Figure 1. We also examined the opposite scenario: EPS that were updated only after the eye has already landed at the new fixation position.

We constructed a range of synthetic eye-position signals, each with a different “target lag,” defined as the time interval between the sigmoid step of the regression target and that of the actual eye. A unique set of pooling weights was estimated for the output variables ($\hat{X}$ and $\hat{Y}$) for each synthetic EPS (see Materials and Methods).

**Figure 4:**
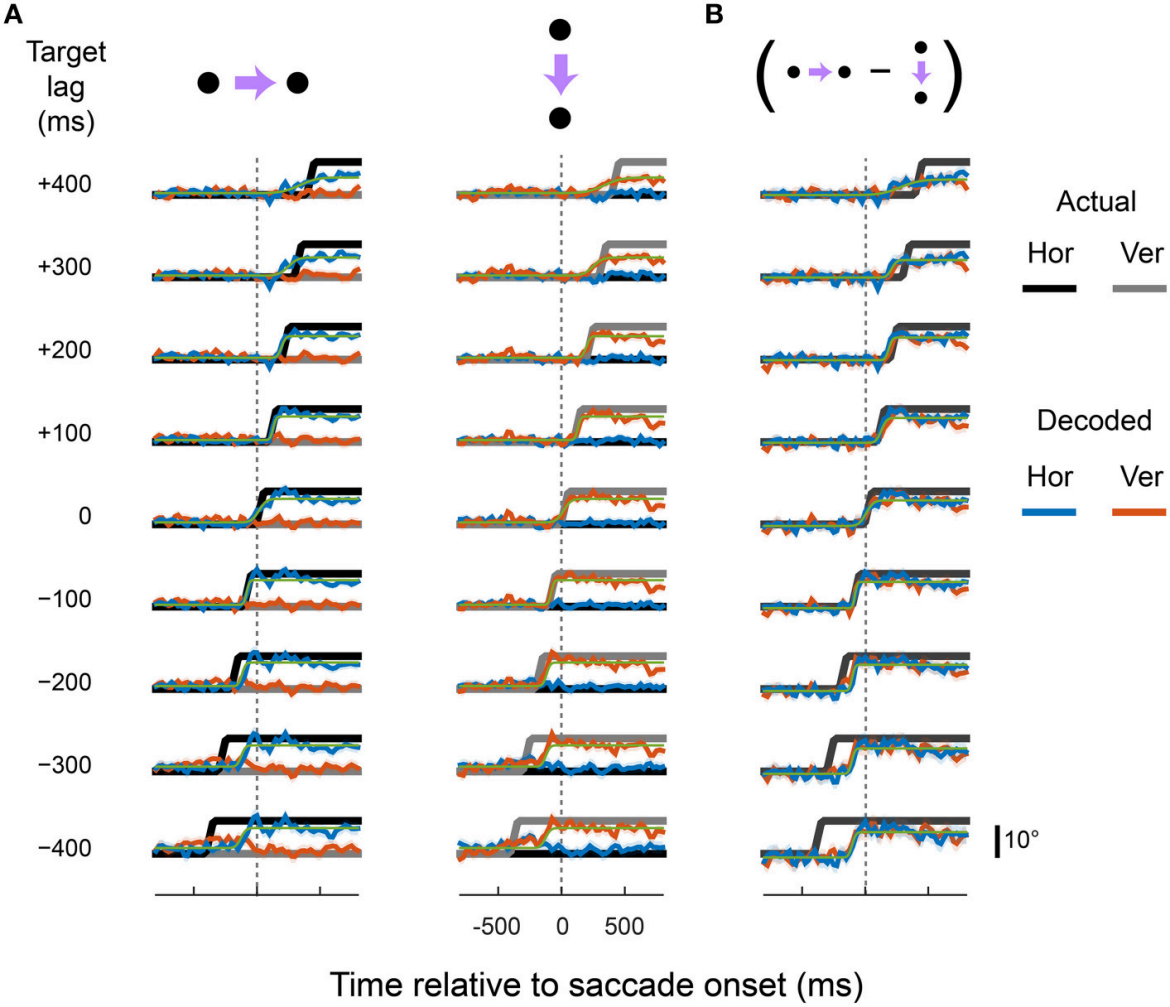
**Predictive and delayed representations of eye position co-exist in area LIP**. The decoder optimized pooling weights to best match a synthetic eye-position signal (black and gray) that shifted to the new fixation position either before (negative target lags) or after (positive target lags) the actual eye movement. **(A)** Each row shows the output of the decoder (in the same format as Figure 3) for a given target lag, using a fixed set of pooling weights for both rightward and downward saccades (columns). The saccade channels were fit with a sigmoid (green curve) to parameterize the lag and saccade duration of the decoded eye-position signal (values shown in **Figure 8**). **(B)** The direction-specific components of the decoder time courses shown in **(A)**, calculated as the difference between rightward and downward saccades for each channel (the vertical channel is plotted with a sign-flip for visualization).

**Figure 5:**
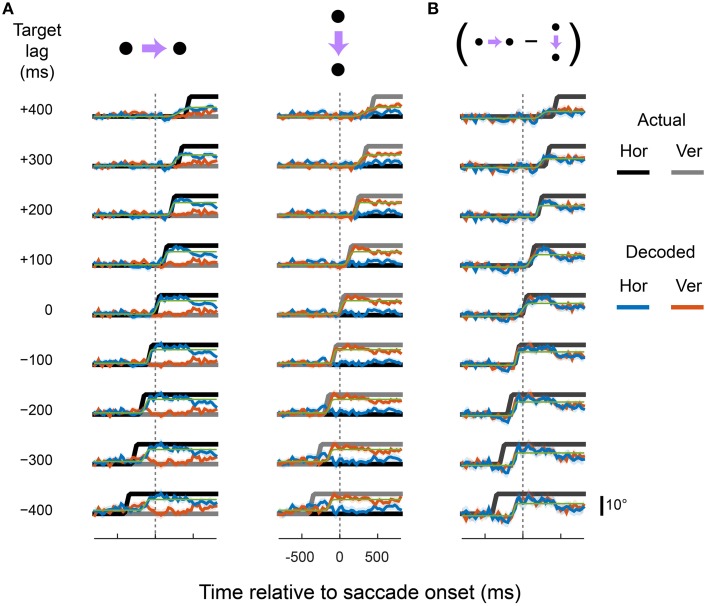
**Predictive and delayed representations of eye position co-exist in area VIP**. Figure is in the same format as Figure 4.

**Figure 6:**
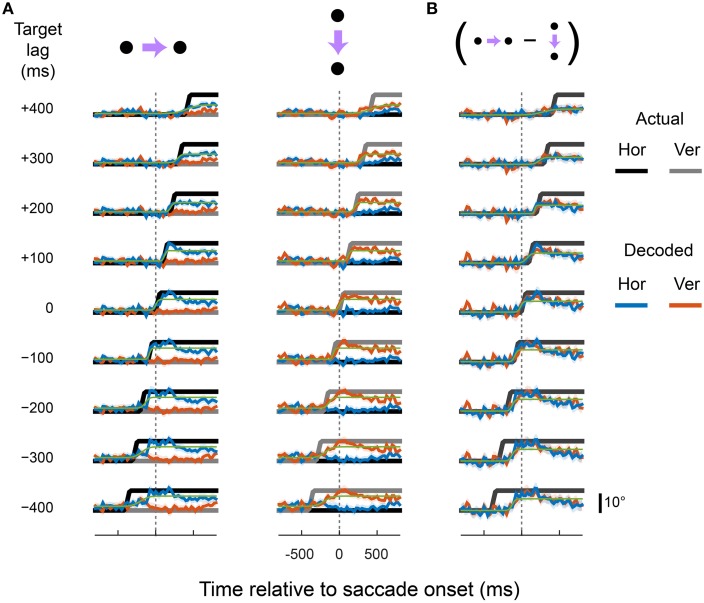
**Predictive and delayed representations of eye position co-exist in areas MT and MST**. Figure is in the same format as Figure 4.

Figures 4–6 show the output of the $\hat{X}$ and $\hat{Y}$ units for a range of lags (from those that lead the eye by up to 400 ms to those that lag by up to 400 ms), separately for each cortical region. The target signals are also shown. The recorded neurons supported a diverse range of dynamics, including representations of eye movements that were either fully predictive or delayed. For example, the output of the $\hat{X}$ and $\hat{Y}$ units closely matched the target signal that lead the actual eye by 100 ms, in both rightward and downward saccade conditions and in all three cortical areas. This representation of the eye reached the new fixation position ~50 ms before the animal's eyes had actually begun to move. Remarkably, with a different set of pooling weights, the same neurons were equally able to represent an eye-position signal that was delayed by 100 ms relative to the actual eye.

**Figure 7:**
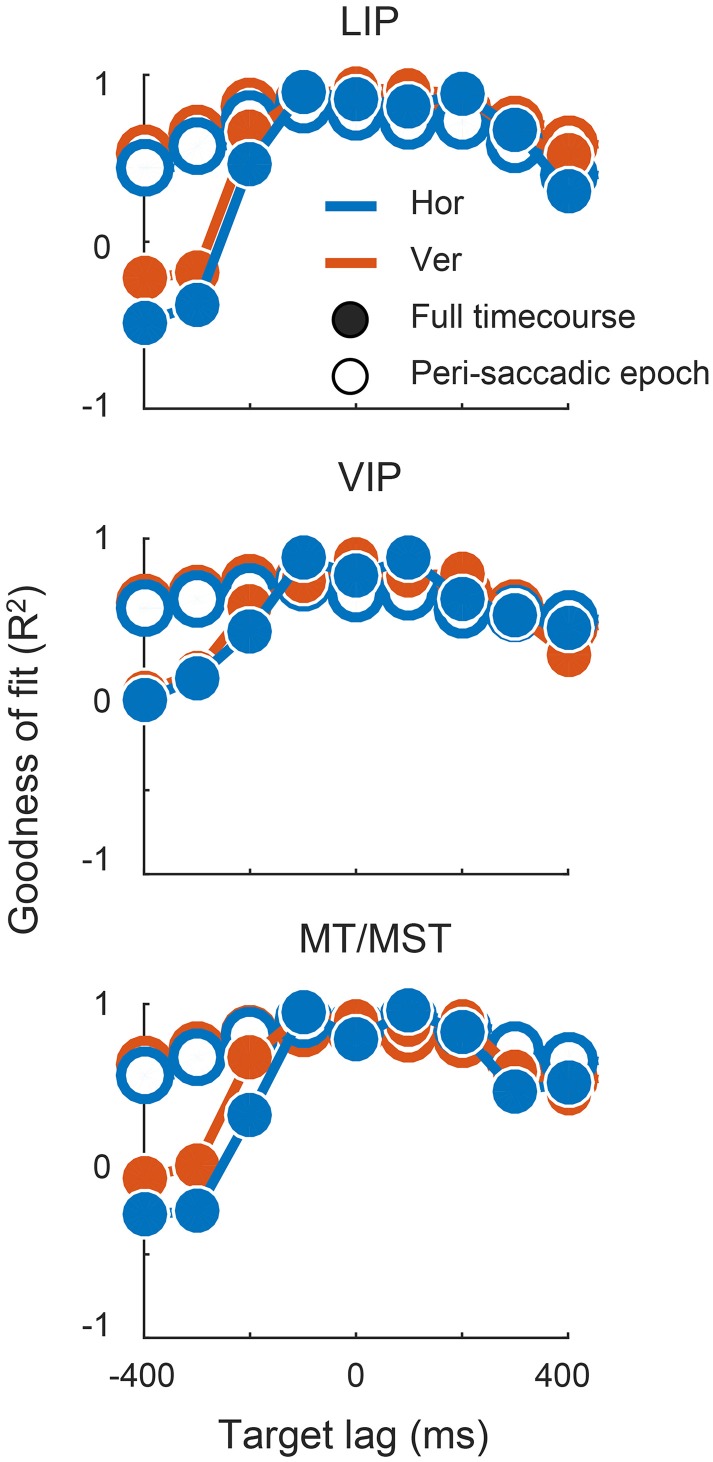
**The decoder output provided a good match to the synthetic eye-position signals across a range of lags**. Plots show the for each channel at each target lag, quantified as the amount of variance in the target signal that was accounted for by the decoder output. Fit error was calculated across the full-time course, and over a restricted “peri-saccadic” interval centered on the time of the saccade in the synthetic target signal (not the time of the animal's actual eye movement).

There were, however, limits to the neurons' ability to represent time-lagged eye-position signals. Target signals that were updated more than 100 ms before or 200 ms after the saccade were fit poorly by the linear read-out model. In those cases, the outputs either drifted slowly toward the post-saccadic eye position (e.g., for target lags > 300 ms), or showed a step-like transition at a time that did not match that of the target signal. Figure 7 shows the goodness of fit (*R*^2^) for all target lags, plotted separately for each cortical region. Fit measures are provided for the full time courses, and for when the calculations were restricted to the [−100; 100] ms “peri-saccadic” interval of the target signal (that is, around the time the target signal was updated). These goodness of fit measures suggest that neurons supported accurate representations across a range of time-lagged signals, from those that led the eye by 100 ms through to those that lagged by as much as 200 ms.

To quantify the dynamics of the decoders, we fit cumulative Gaussians to the predicted time courses (shown by the green curves in Figures 4–6). The inflection point of the fit (i.e., the Gaussian's mean) represented the achieved signal lag, and the interval between the 1st and 99th percentile of the Gaussian was used to estimate the duration of the predicted saccade. By comparing these measures with the equivalent features of the target signal, we gain insight into the ability of this neuronal population to represent a specific lagging EPS.

**Figure 8:**
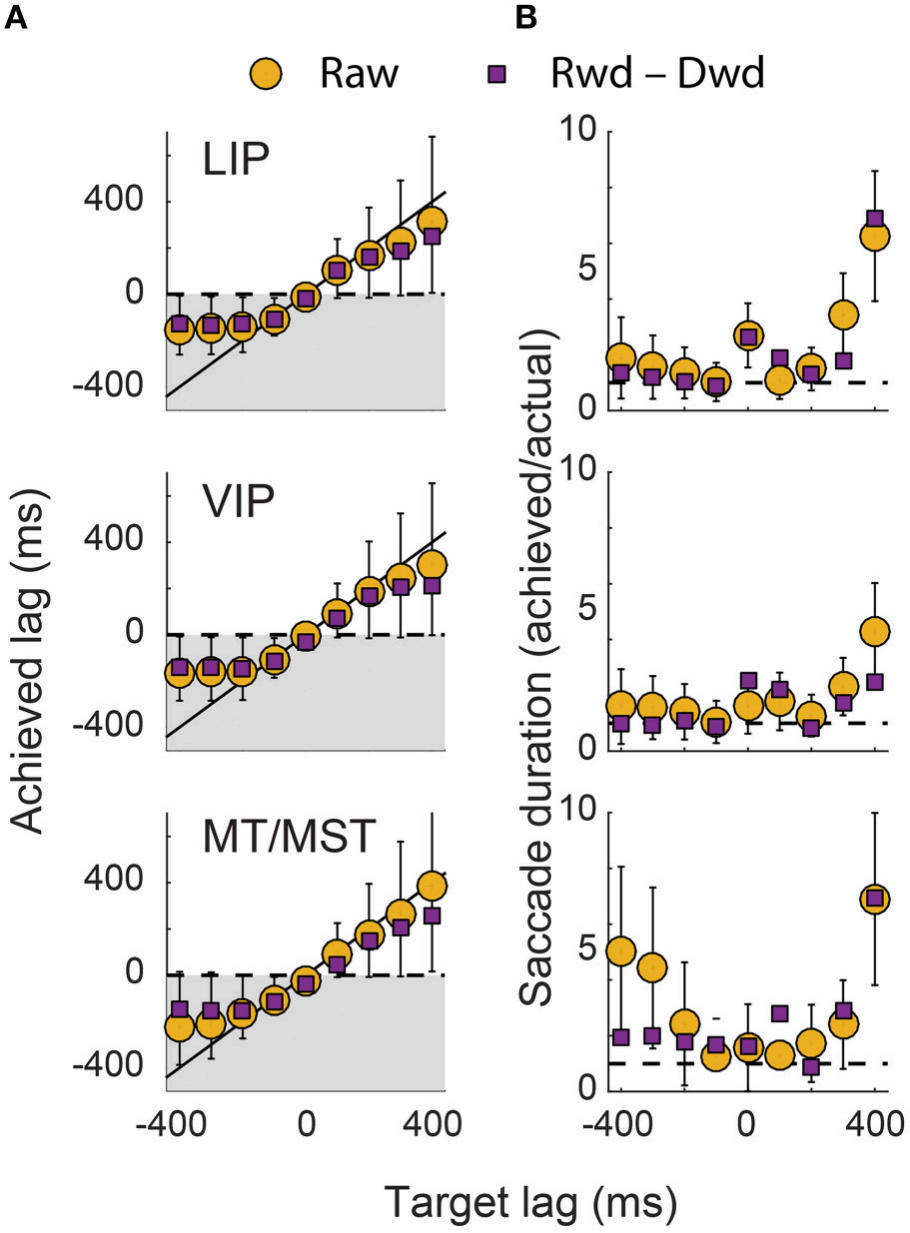
**Dynamics of the decoded eye-position signals. (A)** The lag of the decoded eye-position signals are plotted against those of the target signals. Perfect decoding would result in points along the unity line. Achieved lags that were below zero (shaded region) correspond to representations of the eye that were updated ahead of the actual eye. **(B)** The duration of the saccade represented in the decoder output, expressed as a ratio of the actual saccade duration. Error bars represent 95% confidence intervals, obtained using MATLAB's “nlparci” function and the Jacobian from the sigmoid fits in Figures 4–6. The upper error bars for the +400 ms target lag in **(A)** have been truncated.

Figure 8 shows these lag and duration measures for all target lag conditions, plotted separately for each cortical area. The achieved lags matched those of the target signal for all scenarios except those in which the target signal anticipated the eye by more than 200 ms. The predictive and delayed nature of many of these achieved lags was statistically significant (i.e., those for which the error bars in the figure do not cross zero). For the extreme lags, however, the duration of the represented saccade grossly over-estimated that of the actual eye movement. A good match for both lag and duration was achieved only for lag times between −100 ms and +200 ms, consistent with the goodness of fit measures reported above.

The generally poor performance of the decoders for target signals that led the eye by 300 ms or more was expected. After all, the direction of the impending saccade could not have been known to the neurons earlier than ~213 ms (the average saccade latency) before saccade onset, when the fixation point was displaced. Moreover, the decoders used fixed weights for $\hat{X}$ and $\hat{Y}$ across both saccade direction conditions. In this light, the achieved lags for the target lags of −300 ms and −400 ms were curiously early (<−200 ms). Closer inspection of Figures 4–6, however, reveals the explanation for this effect: the decoder achieved its solution in those cases by allowing the eye-position signal to drift obliquely before the saccade direction was known, as a compromise between the two possible directions. A useful EPS, in contrast, should specify the true future, current, or past eye position, not an amalgam of possible positions.

We therefore asked at what time *direction-specific* changes in the eye position representations emerged. To this end, panel B in Figures 4–6 shows the difference in time courses between the two saccade directions within each channel (i.e., horizontal and vertical). For most target lags, the data recapitulated the dynamics seen in the raw decoder output, confirming that the über-neurons in those cases carried true, direction-specific EPS. For the target lags in which the decoder drifted obliquely in anticipation, however, very different dynamics were observed. Consistent with intuition, direction-specific changes did not emerge until shortly before the onset of the saccade. The timing and duration of these direction-specific transitions were quantified using the same procedure as for the raw time courses (i.e., by fitting cumulative Gaussian functions), and are plotted in Figure 8 as purple squares.

In sum, neurons in the cortical regions we examined supported a continuum of accurate, time-shifted representations of eye movements, including signals that led the eye by as much as 100 ms and lagged by up to 200 ms. Target signals that were updated outside of these bounds were approximated poorly by the über-neurons, reflecting the limits of peri-saccadic information about future and past eye positions.

### How is the labor divided across neurons?

In principle, an über-neuron could match a target signal by assigning approximately equal weights to all neurons, at one extreme, or high weights to one or a small number of neurons and near-zero to the rest, at the other. We examined the sparseness of the decoded representations by evaluating the contribution of each recorded neuron to the über-neurons for each decoder (see Materials and Methods). The analysis was performed only for decoders that provided an adequate fit to the target signal (defined as a total *R*^2^ of greater than 0.75 for the peri-saccadic epoch; see Figure 7).

**Figure 9:**
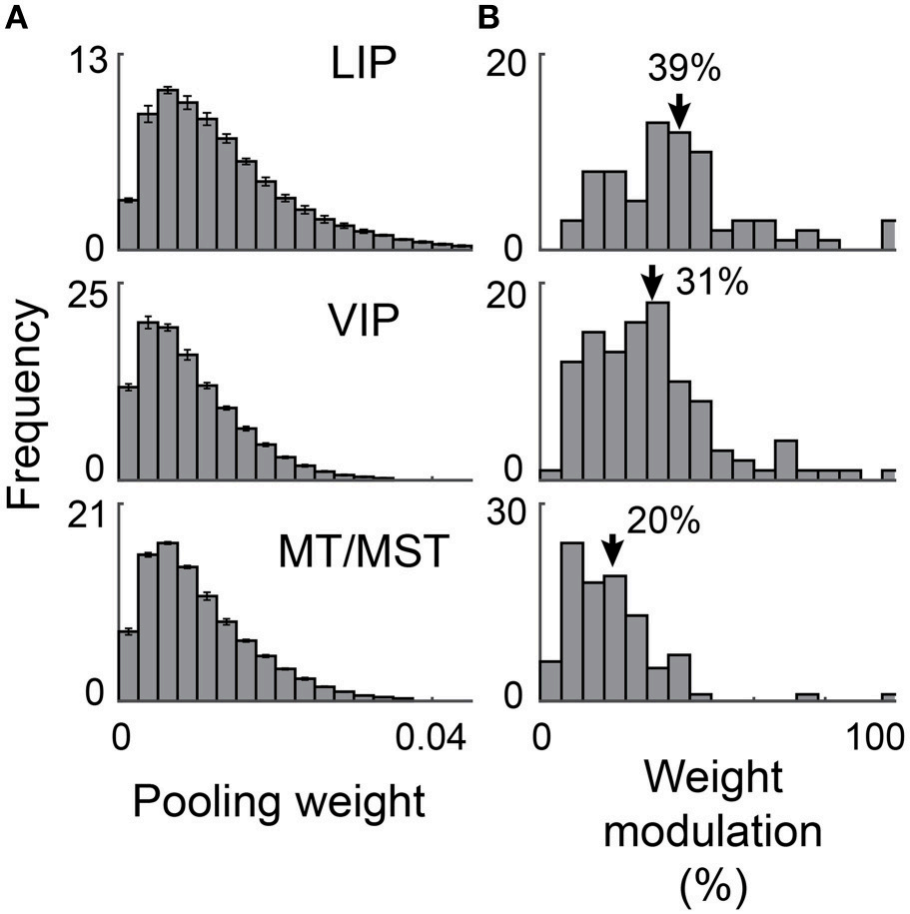
**Distributed population coding of eye position**. We determined how much each neuron contributed to each decoder as a proportion of the total output (“pooling weight”). We also determined how much these single neuron contributions varied across the target lags (“weight modulation”; see Materials and Methods). **(A)** A histogram of pooling weights across neurons was computed for each target lag. The plotted values and error bars are the mean and standard deviation across these histograms. **(B)** Histograms of the weight modulations. Mean modulation values are shown by arrows. This figure shows that most neurons contributed to each decoder, and that different signal lags were achieved using only weak modulations of read-out weights. Both of these properties are compatible with a distributed, not a sparse code.

Figure 9A the distribution of these contributions for each cortical region. The bell-like shape of these distributions indicates that most neurons had intermediate contributions, consistent with broad pooling of activity across the population. The error bars in the figures represent the standard deviation of these distributions across the different target lags. Their small magnitude indicates there was a high degree of consistency, even though the spatiotemporal dynamics of the output varied greatly. This hints that the different decoders were achieved using very similar weights for each recorded neuron. Indeed, the mean contributions for individual neurons were highly correlated across target lag conditions in all three cortical regions (mean correlations across all pairings of lags were 0.88 [STE = 0.03], 0.91 [STE = 0.02], and 0.94 [STE = 0.00], for LIP, VIP, and MT/MST respectively.

To explore this further, we calculated how much the contribution of each neuron varied across the different lags. Specifically, we calculated the difference between each neuron's maximum and minimum contribution and expressed this difference as a percentage of its mean contribution over lags. Figure 9B shows the distribution of these modulation values across the sample for each cortical region. On average, the contributions were modulated by 39% (STE = 3%), 31% (STE = 2%), and 20% (STE = 1.5%) for areas LIP, VIP, and MT/MST respectively. These relatively small differences in weight strength for individual neurons were apparently sufficient to generate the diverse range of time courses evident at the population level (shown in Figures 4–6).

## Discussion

Our analysis shows that neurons in extrastriate and PPC carry a continuum of time-lagged representations of eye position, including predictive, zero-lag, and post dictive signals. These flexible signals were found in all the regions we studied, including those that are not directly involved in saccade planning (VIP, MT/MST). The representations were not superficially evident in the firing rates of single neurons but manifest only when population activity was read out appropriately by artificial downstream neurons (“über-neurons”). With different synaptic weightings, über-neurons carried EPS that shifted toward the new fixation position in sync with the actual eye, led it by up to 100 ms, or lagged behind it by up to 200 ms. These peri-saccadic limits on accurate time-lagged EPS align well with the typical duration of the intersaccadic interval during normal vision (≈300 ms, Ballard et al., 2000).

The 100 ms limitation on the predictive effect is likely determined at least in part by the nature of the instructed saccade paradigm used in this study; given the typical visual latencies in these areas, information about the impending saccade direction could not have been available much earlier. The post-saccadic limit of ~200 ms, however, was not constrained by our experimental paradigm and reflects the fading neural memory of past eye positions in the neural population (or at least that available to a linear decoder).

### Can we record from über-neurons?

In light of our results, one might consider it curious that there are (to our knowledge) no empirical accounts of cortical neurons that exhibit zero-lag or predictive dynamics like our über-neurons. Indeed, Xu et al. (2012) examined the updating of gain-field neurons across saccades and found that firing rate modulations lagged behind the eye by as much as 150 ms. In that study, accurate localization behavior for stimuli presented earlier than this time was interpreted as evidence against the prevailing view of gain-fields and their role in spatial vision. This conclusion, however, was derived from the behavior of a subset of neurons that showed peri-saccadic firing rate modulations that could be understood intuitively in terms of their gain-fields. The remaining neurons—which made up roughly a third of their sample—showed more complex perisaccadic behavior and were labeled as “inconsistent” cells. Our PCA results confirm the considerable heterogeneity and complexity of perisaccadic behavior in LIP and extend it to MT, MST, and VIP. Our population decoding approach, however, treated this diversity as a potent source of information about eye movements and revealed that accurate eye position information is available throughout the peri-saccadic epoch.

In our view, single neurons are unlikely to be devoted exclusively to the purpose of representing eye position. Therefore, a search for über-neurons with dynamics like those reported here would likely be fruitless. Neurons multiplex a large variety of signals in their firing rates and participate in a multitude of functions simultaneously. LIP firing rates, for example, are influenced by visual (Colby et al., 1996), motor (Barash et al., 1991; Snyder et al., 1997), attentional (Bisley and Goldberg, 2003), choice (Roitman and Shadlen, 2002), and reward-related (Louie and Glimcher, 2010) variables, all of which vary over time (presumably contributing to their “inconsistent” perisaccadic behavior). These extraneous contributions to a neuron's output would add to, and potentially mask, step-like inputs related to eye position like those in Figures 3–6. Moreover, in our decoding analysis, it was purely a matter of convenience that we converged the summed neural activity onto a single output unit. We could have equally distributed this pooled signal across a large number of output units with no loss of information; and yet, doing so would render its step-like nature essentially invisible to the naked eye at the level of single neurons.

Nevertheless, we find the concept of a linear über-neuron appealing because it shows that the decoding we perform is not complex and could be achieved in a monosynaptic computation in the brain. Further, it provides a convenient way to visualize the EPS, even though the brain might access these high-dimensional population codes in smarter ways than we can currently imagine. EPS are ubiquitous throughout cortex (V1: Trotter and Celebrini, 1999; V3A: Galletti and Battaglini, 1989; V4: Bremmer, 2000; V6A: Breveglieri et al., 2012; V6: Galletti et al., 1995; frontal eye fields: Cassanello and Ferrera, 2007; premotor areas: Boussaoud et al., 1998). In principle, each of these areas may contain similarly flexible representations that support saccade-invariant computations. Testing these hypotheses is an important direction for future work that will contribute to our understanding of distributed computation in the brain.

### Categorical vs. metric decoding

In some respects, our conclusions mirror those of a recent study that used probabilistic population decoding to examine eye-position signals in LIP (Graf and Andersen, 2014). Graf and Andersen also showed that LIP neurons carry information about past, present, and future eye positions. There are, however, important differences between their study and ours.

First, their decoders chose among a coarse experimental grid of possible eye positions (i.e., the decoder was a classifier), whereas ours estimated eye position as a continuous variable between a start and end position (i.e., it has a metric). The constraints of workable experimental designs result in complex conditional probabilities between eye movement parameters (e.g., the rightmost eye positions in a grid can only be reached by rightward saccades). A classifier can exploit these contingencies to achieve above chance decoding performance, even though they are unlikely to be useful in real life. A metric decoder is more in line with the type of signal the brain requires for spatial processing, and also has the practical advantage that one can study systematic errors on a fine spatial scale (e.g., Morris et al., 2012, 2013).

Second, Graf and Andersen constructed a new decoder for each time window relative to saccade onset. For instance, to decode future eye position, they constructed different Bayesian classifiers based on firing rates in time windows before, during, and after the saccade. This approach accurately quantifies the information available in each of these epochs, but it is not clear how the brain might implement this kind of read-out in which the decoder changes over time. Xu et al. (2012) raised similar reservations in considering how the brain might switch between representations built from their “consistent” and “inconsistent” cells (which they treated as separate populations). In our study, however, the pooling weights were held constant over time and across saccade directions. This allows us to interpret the decoder output as a proxy for signals that could be computed easily in the brain and motivates our coining of the über-neuron term; these signals are not just a quantification of information that is, in principle, available in the population, but information that can be extracted in a monosynaptic step.

Third, Graf and Andersen employed a memory-saccade paradigm, whereas our animals made immediate saccades to visual cues. The memory-saccade paradigm has the advantage that it can dissociate saccade planning from saccade execution and visual signals. Indeed, we cannot rule out the possibility that at least some of the predictive information about future eye positions in our study is derived from visually-evoked activity. Visual influences, however, would be maximal immediately after the onset of the target (i.e., ~130 ms before saccade onset, based on the mean visual latency of 80 ms); and yet, these neurons supported predictive signals that remained stable at this time and were only updated later (e.g., the −100 ms lag reported here, as well as any lag between −100 ms and 0 [data not shown]). This suggests that visual influences are unlikely to account for the predictive eye-position signals reported here. Applying our approach to experiments with stable visual displays or memory-guided saccades is needed to fully resolve this question.

In Graf and Andersen's study, the future eye position could be decoded reliably from the moment the target had been specified, even though the saccade itself did not occur until more than half a second later. This suggests that the decoder was able to use the current eye position and direction-selective planning activity to infer the future eye position (similar to modeling studies: Ziesche and Hamker, 2011, 2014; Schneegans and Schöner, 2012). The absence of clear modulations in this performance at the time of saccade execution (their Figure 2) is consistent with this notion. Given the differences in analysis and the coarseness of this categorical approach (discussed above), this result does not necessarily extend to the metric decoding we advocate here, and future work is needed to address this issue. Similarly, our paradigm included only two saccade directions, potentially allowing the decoder to exploit directional preparatory signals that might be weaker during normal, unconstrained vision. It remains to be seen whether a simple linear read-out rule like that reported here generalizes to these other scenarios. Nevertheless, it's likely that our decoder—and the brain—exploits direction-selective modulations of activity before and during saccades to complement potentially slower inputs that explicitly carried eye position information (e.g., proprioception, Wang et al., 2007; Schneegans and Schöner, 2012; Xu et al., 2012; Ziesche and Hamker, 2014).

A fourth difference between our study and that of Graf and Andersen is that their decoder provided estimates of *absolute* eye position (i.e., the direction of the eyes in the head), whereas ours provides an estimate of eye position *relative to its starting position*. We have shown previously using the same data-set, however, that these neurons also provide an accurate and precise representation of absolute eye position during fixation (albeit with a non-linear read-out approach). This suggests that the current results can nevertheless be interpreted in absolute terms by assuming a simple combination of the two (relative and absolute) signals.

Finally, Graf and Andersen observed that most of the eye position information used by the decoder was obtained from a small subset of neurons (~20), whereas our über-neurons pooled broadly across neurons. This apparent discrepancy perhaps reflects the different emphasis in the studies. Our decoder assigns weights to neurons for their ability to provide consistent information on the full spatiotemporal dynamics of a saccade. This constraint resulted in a distributed code. Graf and Andersen, however, constructed a new classifier in each 250 ms time window; it would seem that a small subset of neurons (i.e., a sparse code) carries most of the information in each of these windows.

### Peri-saccadic mislocalization

In Morris et al. (2012), we showed that these neurons carry a damped representation of eye position that could explain why observers misperceive the location of stimuli that are flashed around the time of a saccade (Honda, 1991; Dassonville et al., 1992). In that study, we divided the sample into two sub-populations based on the slope of their gain-field and the direction of the saccade; one contained the neurons that were moving from a low-to-high firing rate, and the other contained those making the opposite transition. Using the difference in activity between groups as a representation of eye position, we observed damped eye positions signal dynamics, such that the decoded eye moved toward the saccade end-point predictively but did not finalize its transition until after the real eye had landed.

Although it was not framed as such, that analysis was in some respects a rudimentary version of the population decoding reported here; that is, neurons in the two sub-populations received binary weights of 1 and −1 (though these weights had to be re-assigned for every saccade direction, unlike in the current study). Our current findings show that given the freedom to choose graded weights, a decoder can generate a near-veridical representation of the eye, as well as a range of time-lagged variants.

This raises the question why the perceptual system makes errors in the context of this well-established experimental paradigm. One explanation is that even if eye position information is veridical, information on the location of the stimulus on the retina may not be (Krekelberg et al., 2003). In addition, however, we suggest that although a suitable EPS is available, the visual system may not have ready access to the required read-out under such artificial conditions. Objects rarely appear and disappear around the time of saccades during natural vision. As such, the read-out mechanisms that govern localization should be optimized for the representation of objects during fixation (Niemeier et al., 2003). When forced to locate peri-saccadic objects, sub-optimal read-out of eye position may blend EPS with different lags, leading to a damped net representation of the eye.

We tested this idea informally by omitting the peri-saccadic epoch (i.e., 100 ms either side of the saccade) during the estimation of weights for each neuron, such that the decoder is optimized for representing eye position during fixation. We then predicted the EPS for all times, including the perisaccadic window. As expected, this EPS was clearly damped, similar to the one we reported previously, and qualitatively consistent with perisaccadic mislocalization. Of course, there are likely other factors at play, such as uncertainty regarding the temporal onset of the visual flash (Boucher et al., 2001), visual latencies and persistence (Pola, 2004), and the influence of spatial references (Lappe et al., 2000).

### What are these signals good for?

Although perhaps counter-intuitive, a perfect, zero-lag representation of eye position may in general be no more useful to the brain than a range of other time-lagged signals, such as those that are updated before or after eye movement. In the standard gain-field model of spatial processing, the co-existence of eye-centered visual information and eye-position signals gives rise to an implicit, head-centered representation of visual space (Andersen et al., 1985; Zipser and Andersen, 1988; Pouget and Sejnowski, 1997; Bremmer et al., 1999). This representation is thought to provide a basis for goal-directed actions, such as reaching and navigation, as well as multisensory integration (Pouget et al., 2002). For head-centered spatial information to remain accurate across eye movements, however, both input types—visual and eye position—need to be updated in synchrony. It takes time for reafferent visual input from each new fixation to reach cortex, suggesting that a *delayed* EPS would likely be optimal (Teichert et al., 2010).

The required delay, however, would vary across cortical areas in accordance with differences in visual latencies. Although this variation is fairly modest in traditional measurements during fixation (tens of milliseconds, Schmolesky et al., 1998), experiments involving eye movements have revealed transient changes in spatial selectivity that can be considered to give neurons negative or unusually short visual latencies (Duhamel et al., 1992). This phenomenon, known as “predictive remapping,” is widespread, particularly in higher cortical areas (Nakamura and Colby, 2002), and is characterized by neurons that update eye-centered representations of visual space in *advance* of a saccade. Such neurons would seem to require a predictive representation of eye position to maintain visual stability.

Taken together, these considerations suggest that a single, global EPS might be insufficient to support stable vision. Our results show that through appropriate synaptic weighting, an EPS can be tailor-made for a given neuron or population to ensure that it is notified of changes in eye position only at the suitable time. That is, the cortex could be furnished with an essentially infinite number of different EPS, all achieved through unique pooling of signals. Local computations, therefore, could incorporate information about past, current, and future eye positions simultaneously. This could allow, for example, self-induced changes in sensory representation to be dealt with differently to those caused by true changes in the outside world (Crapse and Sommer, 2012; Ziesche and Hamker, 2014).

Remarkably, our analysis of pooling weights suggests that profoundly different time courses can be achieved through modest local adjustments (20–40% on average) to a coarse and universal weighting template. To an extent, this is not surprising, given that the target signals used here differed only in the timing of the saccade representation and had in common the extensive fixation intervals (the correlation between pairs of target signals was between 0.35 and 0.91; mean = 0.68). Nevertheless, the profound global effects of such subtle changes to the network provides a striking example of the powerful yet obscure nature of distributed population codes. Further, it emphasizes the need for population-level analysis techniques to unmask the underlying representations.

## Author contributions

AM, FB, and BK designed and performed the research, and wrote the paper; AM and BK contributed analytic tools and analyzed the data.

### Conflict of interest statement

The authors declare that the research was conducted in the absence of any commercial or financial relationships that could be construed as a potential conflict of interest.
